# Emergence of a Code in the Polymerization of Amino Acids along RNA Templates

**DOI:** 10.1371/journal.pone.0005773

**Published:** 2009-06-03

**Authors:** Jean Lehmann, Michel Cibils, Albert Libchaber

**Affiliations:** 1 Center for Studies in Physics and Biology, The Rockefeller University, New York, New York, United States of America; 2 Section de Mathématiques, Ecole Polytechnique Fédérale de Lausanne, Lausanne, Switzerland; German Cancer Research Center, Germany

## Abstract

The origin of the genetic code in the context of an RNA world is a major problem in the field of biophysical chemistry. In this paper, we describe how the polymerization of amino acids along RNA templates can be affected by the properties of both molecules. Considering a system without enzymes, in which the tRNAs (the translation adaptors) are *not* loaded selectively with amino acids, we show that an elementary translation governed by a Michaelis-Menten type of kinetics can follow different polymerization regimes: random polymerization, homopolymerization and coded polymerization. The regime under which the system is running is set by the relative concentrations of the amino acids and the kinetic constants involved. We point out that the coding regime can naturally occur under prebiotic conditions. It generates partially coded proteins through a mechanism which is remarkably robust against non-specific interactions (mismatches) between the adaptors and the RNA template. Features of the genetic code support the existence of this early translation system.

## Introduction

**Figure 1:**
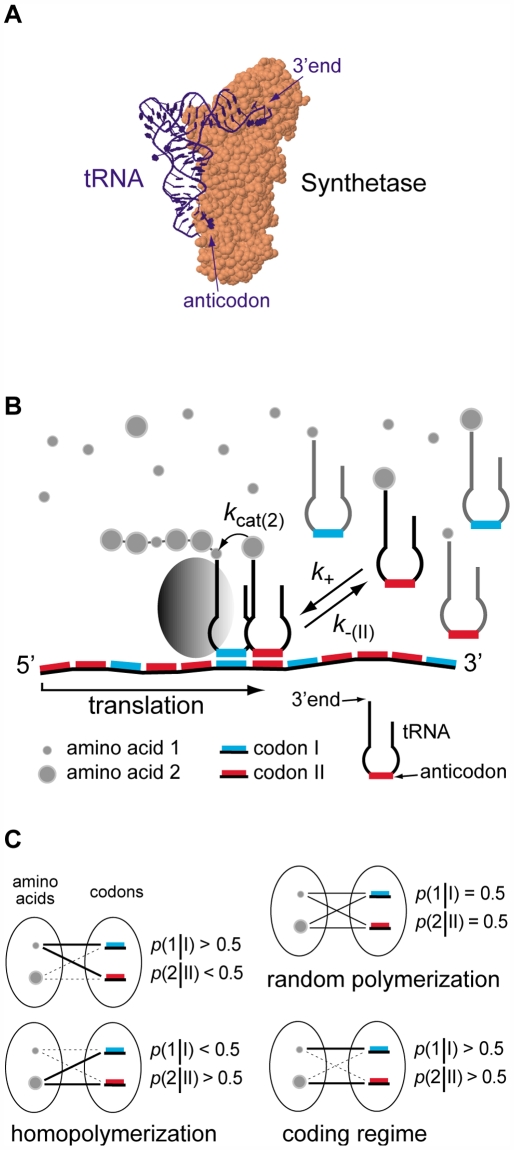
Model of the elementary translation system. (a) Synthetase with cognate tRNA (structure 1ZJW from *Protein Data Bank*). (b) Kinetic scheme of the elementary translation process. Two types of tRNAs (complementary to two template codons) are *unselectively* loaded with two types of amino acids (the rates of loading are only concentration-dependent). The grey rugby ball is a stabilizing cofactor (see text for explanations). (c) Characteristic regimes of the polymerization process.

A major issue about the origin of the genetic system is to understand how coding rules were generated before the appearance of a family of coded enzymes, the aminoacyl-tRNA synthetases. Each of these ∼20 different enzymes has a binding pocket specific for one of the 20 encoded amino acids, and also displays an affinity for a particular tRNA, the adaptor for translation [Fig. 1(a)]. These adaptors are characterized by their anticodons, a triplet of base located on a loop. The synthetases establish the code by attaching specific amino acids onto the 3′ ends of their corresponding tRNAs, a two-step process called aminoacylation [1]. The first step (activation) involves an ATP, and leads to the formation of a highly reactive intermediate, aa–AMP (aa = amino acid). The second step consists of the transfer of the amino acid from AMP onto the 3′ end of the tRNA. Those tRNAs can subsequently participate in the translation of RNA templates, during which codons about to be translated are tested by the anticodons of incoming tRNAs. When anticodon-codon complementarity occurs, an amino acid is added onto the nascent protein through the formation of a new peptide bond [2].

How could a translation system operate in the absence of the synthetases? Recent works have shown that particular RNA stem-loops of ∼25 bases can self-catalyze the covalent binding of amino acids onto their own 3′ ends [3], [4]. These RNAs however require aa–AMP as a substrate because they cannot manage the activation step in their present form. In addition, they show little specificity for the amino acids, raising the question of how a code could be generated by them. Some answers will likely be provided by the activation step if possible to implement on these small RNAs. This issue is not examined in the present paper.

Based on an earlier investigation [5], the present analysis shows that the translation process itself can contribute to the establishment of coding rules. Consider an elementary translation system constituted by RNA templates made up of two types of codons {I, II}, tRNAs with anticodons complementary to these codons, and two types of amino acids {1, 2}. Suppose that the tRNAs are *not* selectively loaded with amino acids (i.e. the rates of loading only depend on the relative concentrations of the amino acids). Our analysis shows that it is possible to observe a coded polymerization. We calculate the probability of codon I being translated by amino acid 1 and the probability of codon II being translated by amino acid 2, the coding regime occurring when both probabilities are *simultaneously* higher than 0.5. These probabilities are functions of the anticodon-codon association and dissociation rate constants, the amino acids concentrations and their respective kinetic constants of peptide bond formation. One general configuration allows a coding regime to occur: the amino acid with the slow kinetics (i.e. the “slow” amino acid) is more concentrated in solution than the “fast” amino acid. Given two appropriate codons, the competition for the translation of the codon dissociating quickly from its cognate tRNA (i.e. the “weak” codon) is won by the fast amino acid. As for the “strong” codon, for which the amino acid kinetics are equal or higher than the anticodon-codon dissociation rate constant, the higher concentration of the slow amino acid makes it a better competitor in that case. Although other types of polymerization are possible, we show that this coding regime is favored under prebiotic conditions. It is furthermore remarkably robust against anticodon-codon mismatches. We conclude our analysis by showing that this model can naturally be implemented by a system of four codons and four amino acids thought to be a plausible original genetic code.

## Results

### Model

Let us consider two small RNA stem-loops (hereafter called tRNAs) characterized by their anticodons and both capable of loading *as efficiently* two types of amino acids {1, 2} onto their 3′ ends. The rates of aminoacylation will thus simply follow the relative concentrations of these amino acids in solution, [aa_1_] and [aa_2_]. These tRNAs are involved in the translation of an RNA template made up of two types of codons {I, II} complementary to these anticodons. Translation is governed by a Michaelis-Menten type of kinetics: the first step is characterized by anticodon-codon association rate constants (*k*_+_) and dissociation rate constants (*k*_−_), and the second (irreversible) catalytic step is characterized by a kinetic constant *k*_cat_ depending on the amino acids [Fig. 1(b)].

It is assumed that the association rate constants *k*_+_ of all anticodon-codon couples are alike. This situation is expected since it already occurs in the context of anticodon-anticodon interactions [6], which is similar. In a first approximation, only complementary matchings are considered. We therefore have *k*_+_ = constante, *k*_−(I)_ for codon I and *k*_−(II)_ for codon II. As for the kinetics of peptide bond formation (*k*_cat_), an earlier work showed that this variable may strongly depend on the side-chains of the amino acids [5]. Accordingly, two constants (*k*_cat(1)_ and *k*_cat(2)_) are defined.

The model includes a ribosome-like cofactor capable of stabilizing the tRNA carrying the nascent protein on the template [Fig. 1(b)]. Although our analysis may not clarify the molecular origin of this cofactor, two of its properties can be specified, which are required to validate our conclusions:

1. The cofactor does not have a catalytic site for peptide bond formation which could minimize the side-chain effect mentioned above. Modern ribosomes have a catalytic site, the peptidyl-transferase center [7]. It is still not clear to us how this specialized part of the ribosome manages the different side-chains, although the problem has already been considered [8].
2. The cofactor does not have a decoding center. This evolved structure on the small subunit of modern ribosomes allows for an increase in the fidelity of anticodon-codon recognition [9]. This structure is inconsistent with the simplicity of the kinetic scheme described here [Fig. 1(b)]. Our model is therefore in agreement with the hypothesis that the original ribosome was only made up of the large subunit [10].

Overall, the cofactor considered here is *a priori* simple since it does not have specialized parts. Avoiding a break in the continuity of the evolutionary process implies that it was already made up of RNA.

Considering now the dynamic of the translation process, it is assumed for simplicity that the relative concentrations of the aminoacyl-tRNAs remain constant over time. This can be guaranteed by the reversibility of the aminoacylation process [1], which will prevent the accumulation of aminoacyl-tRNAs unfit for translation.

With the above hypotheses, let us define the probability 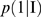 of codon I being translated by amino acid 1:
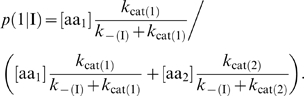
(1)One can similarly write 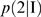, and verify that the normalization condition 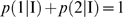 is satisfied. It is convenient for the analysis to relate the different kinetic constants and concentrations with ratios. We denote
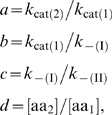
by defining the kinetic constants in order to have *a*≥1 and *c*≤1. Then, expression (1) becomes
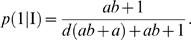
(2)Similarly, the probability of codon II being translated by amino acid 2 is
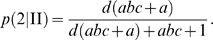
(3)


Typical configurations of (2) and (3) are shown in Fig 1(c), which groups together the main possible outcomes of the polymerization process: random polymerization, homopolymerization, and coding regime. We are particularly interested in the case when this system displays some homogenous coding properties Σ, defined as
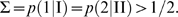
(4)


It can be shown that there is only one solution to the coding problem (i.e. 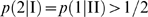 cannot occur).

A few general statements can be made about relation (4). With *a*, *b*, *c*, *d*>0 to be physically meaningful, the coding regime Σ cannot be observed if the amino acids are characterized by identical *k*_cat_ (*a* = 1) or if they are at the same relative concentrations (*d* = 1). In both cases, the only possibility is 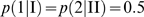 and *a* = *d* = 1. If both codons I and II display identical *k*_−_ (*c* = 1), we also get 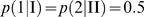, and 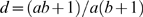.

### Amino acid requirements for the coding regime

An examination of expressions (2) and (3) when *b* varies within the interval ]0, ∞[shows that the coding regime (4) can be satisfied only if *c*<1 and
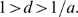
(5)


Condition (5) signifies that *the amino acid with the highest k_cat_ must be less concentrated in solution than the other amino acid*. *This relative concentration must still be higher than 1/a*.

**Figure 2:**
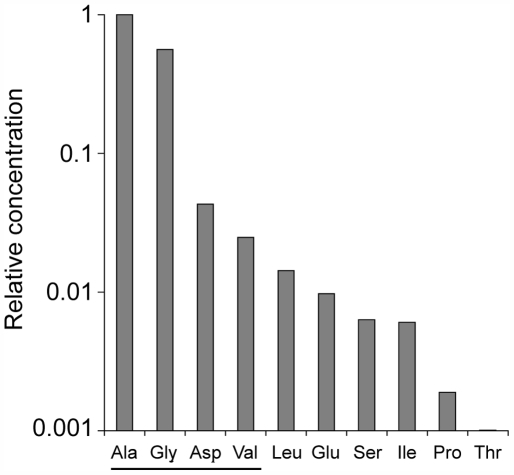
Relative abundance of primitive amino acids. Relative abundance of the 10 most frequent amino acids of the genetic code synthesized in an experiment thought to reproduce the conditions of the prebiotic Earth. Graph established from the data of Table 3 in ref. [11].

**Figure 3:**
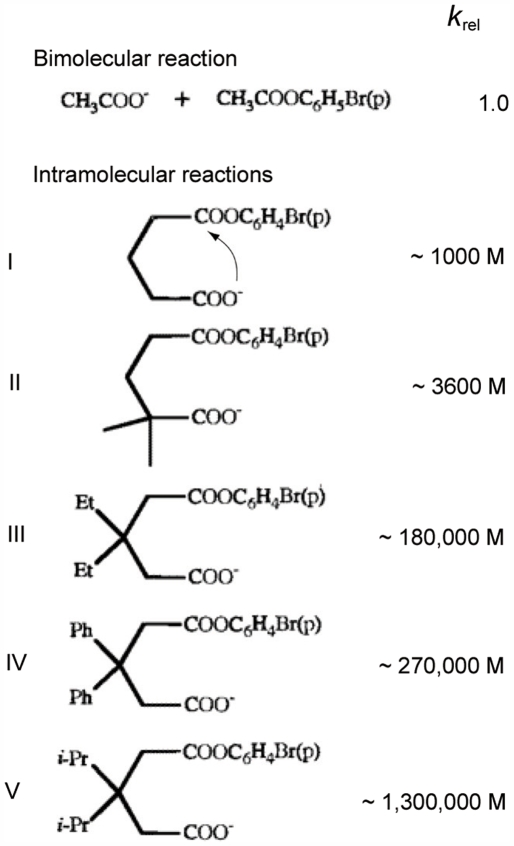
Effect of the local environment on a reaction rate. Relative reaction rates (*k*_rel_) of a bimolecular reaction and some corresponding intramolecular reactions. In the intramolecular systems, the nucleophilic attack (indicated by a small arrow in compound I) leads to the cyclization of the compounds. Adapted from ref. [12].

**Figure 4:**
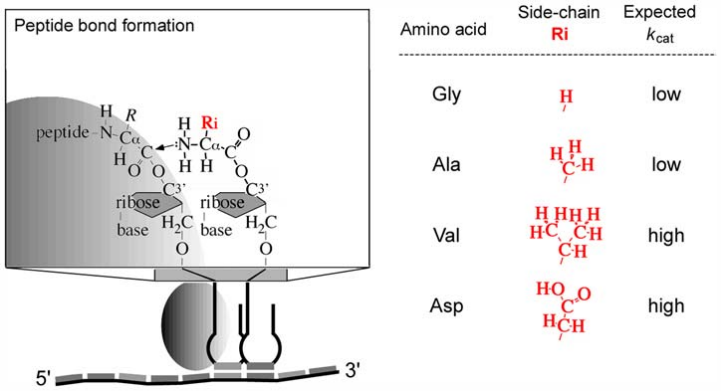
Amino acids side-chains and kinetics of peptide bond formation. Elementary translation and expected effect of the side-chains of alanine, glycine, aspartic acid and valine on the kinetics of peptide bond formation (*k*_cat_) (discussed in the text).

To check whether condition (5) could be reasonably fulfilled at the origin of Life, let us consider some results of the well-known prebiotic synthesis experiments conducted by Miller [11], which revealed what amino acids of the genetic code are the easiest to be generated. The histogram of Fig. 2 shows that glycine and alanine display a similar abundance, and are about one order of magnitude more frequent than the next two amino acids, aspartic acid and valine. Considering now the chemical step, how may each of these amino acids affect the probability of peptide bond formation (*k*_cat_)? Studies of intramolecular reactions [12]–[14] show that the size of the group(s) of atoms bound to the carbon in position 1 or 2 after a nucleophile is usually very critical for a reaction rate. When hydrogens are substituted with a dimethyl, the relative reaction rate (*k*_rel_) may not appreciably change, although the result depends on the system and the position of the substitution [14]. When these substitutions involve bulkier groups (such as diethyl groups), a sudden jump of at least two orders of magnitude of *k*_rel_ is typically observed (Fig. 3). Bulky substituents restrict rotation around bonds, which contributes to the localization of the nucleophile (entropic effect) [13]. These data suggest that glycine and alanine may be characterized by similar *k*_cat_, but that a much higher *k*_cat_ is expected for aspartic acid and valine (Fig. 4). Restricting our analysis to the four most abundant amino acids, the above considerations show that two categories may be established: {Ala, Gly} and {Val, Asp}. Each of these categories comprises amino acids that are similar with respect to *k*_cat_ and concentration. This degeneracy is examined further below. If we assume (as suggested by the above data) that these two categories are related by *a*∼100 and *d*∼0.1, we can conclude that condition (5) may be fulfilled in some primitive environment.

### Codons for the coding regime

Let us now examine how well any two codons (related by *c* = *k*_−(I)_/*k*_−(II)_) can satisfy relation (4). To a low value of *c* corresponds a high value of Σ (see below). The ΔG_0_ values of all complementary anticodon-codon interactions of the genetic code span approximately from −2 to −6 kcal mol^−1^ at T = 310 K [5]. These estimates refer to crude anticodon-codon interactions: they do neither include additional ΔG_0_ contributions occurring within modern ribosomes [9], nor the effect of tRNA anticodon loop refinement taking place in the present-day genetic system [15]–[16]. With *K_eq_* = *k*_+_/*k*_−_ = exp(−ΔG_0_/RT), and with *k*_+_ = constant (see above), the lowest value of *c* lies somewhere between 10^−2^ and 10^−3^.

**Figure 5:**
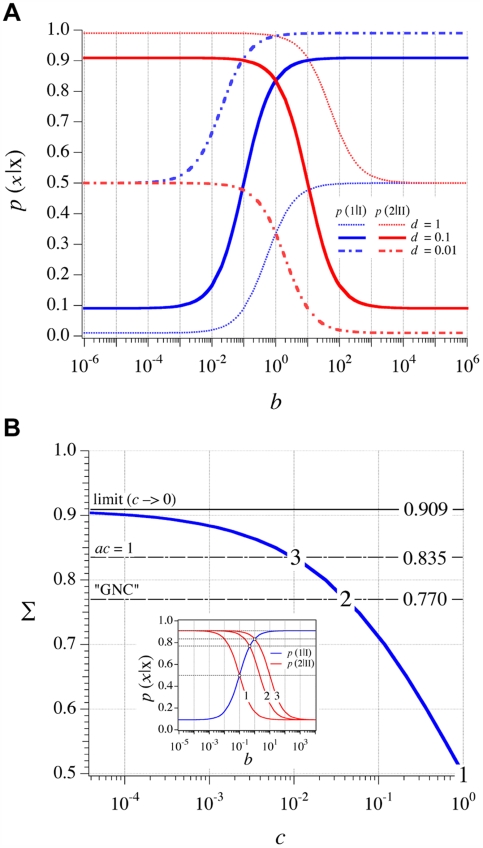
Regimes of the polymerization process. (a) Effect of the value of the ratio *k*_cat(1)_/*k*_−(I)_ on the polymerization process: *p*(1|I) and *p*(2|II) as a function of *b* for three significant values of *d* when *a* = 10^2^ and *c* = 10^−2^. When *d* = 1, either homopolymerization of aa_2_ (*b*<1), or random polymerization (*b*≫1) are observed. When *d* = 0.1, a coding regime Σ = 0.835 is observed at the transition between two types of homopolymerization, which occurs at *b* = 1. When *d* = 0.01, either random polymerization (*b*≪1) or homopolymerization of aa_1_ (*b*>1) are observed. (b) Level of coding Σ as a function of *c* when *a* = 10^2^ and *d* = 10^−1^. Three significant values of Σ are indicated. The inset shows a plot similar to (a) for three particular values of *c* (reported from the main graph).

A particular solution of Σ is obtained when *ac* = 1 which is physically relevant: for a given *a*, Σ increases relatively rapidly as *c* decreases between 1 and 1/*a* [see Fig. 5(a–b)]. The turning point *c* = 1/*a* sets up the maximal value of the “easy gains” for Σ, i.e. gains which do not require an unrealistic ΔΔG_0_ value between the two codons. When *c*<1/*a*, Σ increases less and less, until it reaches a maximal value. Furthermore, a simplification in the algebraic treatment of relation (4) occurring when *ac* = 1 (implying *b* = *k*_cat(1)_/*k*_−(I)_ = *k*_cat(2)_/*k*_−(II)_) allows us to determine that 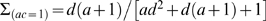 has a maximum with respect to *d* when 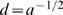. Since 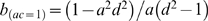, this maximum is also characterized by *b* = 1. This result shows that the above estimates of *a* and *d* are well compatible with a polymerization process in a coding regime. Taking these values (*a* = 10^2^; *d* = 10^−1^) together with *c* = 10^−2^, one gets 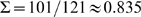.

Fig. 5(a) shows 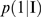 and 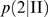 as a function of *b* for three characteristic values of *d*, which allows to graphically check our results with the above numerical values. Remarkably, more realistic calculations including the effect of mismatches show that the coding regime is only marginally affected by them (see below), demonstrating the robustness of this coding system.

### Connecting amino acids with codons

The *b* parameter makes a connection between two kinetic constants of different origins: *k*_cat(1)_ is determined by an amino acid (bound to an RNA) while *k*_−(I)_ is determined by RNA. Among the two constants, only *k*_−(I)_ could be tuned by the translation machinery, through the selection of the tRNA anticodon loop. Anticodons of two bases (if structurally possible) are expected to have a high *k*_−(I)_ because of the low anticodon-codon ΔG_0_ and the importance of thermal fluctuations, while the opposite trend is anticipated for larger anticodons. An examination of Fig. 5(a) from low to high values of *b* reveals that a coding regime occurs at *b*∼1, in the transition between two types of homopolymerization. Below *b* = 1, the outcome of polymerization is controlled by *k*_cat_; above this value, it is controlled by the relative concentrations. The transition region is sensitive to small differences in kinetic constants precisely because *k*_cat(1)_≈*k*_−(I)_. The existence of a correlation in the genetic code reflecting a dependence between these two kinetic constants (Fig. 3 in ref. [5]) supports our analysis, and allows us to connects *b*∼1 with 3-nt anticodons, this size being structurally associated with 7-nt loops [17].

### An elementary form of the genetic code

The above analysis shows that Σ∼0.8 is achieved with the two categories {Ala, Gly} and {Val, Asp} when *c* = 10^−2^. Since any two codons of the existing genetic code can be characterized by *c* values much closer to 1 (implying a random polymerization), it can be concluded that an initial coding system with already 64 codons and 20 amino acid could not work. For several reasons, it has been proposed that the original set of anticodons and codons was limited to ^5′^GNC^3′^, where N is U, C, G or A [18]–[20]. Remarkably, these four codons encode the above four amino acids, the two categories {Ala, Gly} and {Val, Asp} being associated resp. with {GCC, GGC} and {GUC, GAC}. Both codons in each category display identical anticodon-codon ΔG_0_ estimates, and the ΔΔG_0_ between the two categories is ∼1.9 kcal mol^−1^ [5], implying a *c* value of ∼0.045.

Fig. 5(b) shows the level of coding Σ as a function of *c* with *a* = 10^2^ and *d* = 10^−1^. In this configuration, the maximal value that Σ can theoretically reach (c→0) is *da*/(*da*+1)≈0.91. The plot shows that Σ is sensitive to small variations of *c* within the region 1–0.01 (i.e. *ac*≥1), where it rapidly increases as *c* decreases. As for the “GNC system” (*c* = 0.045), Σ reaches a value as high as 0.77. Below 0.01, *c* rapidly falls to 0 as Σ tends to its maximal value.

The above analysis still leaves this coding system of four amino acids and four codons with an issue of degeneracy. Considering for instance {Val, Asp} and {GUC, GAC}, one should ask whether a mechanism could specifically assign Val to GUC and Asp to GAC, as it occurs in the modern genetic code. It can be noticed that in each of the two amino acids categories, one amino acid is hydrophobic (Val, Ala) while the other one is hydrophilic (Asp, Gly). This suggests the possibility of a discrimination during the loading of the amino acids on the tRNAs. This loading indeed necessarily implies an intermolecular association, which is usually strongly conditioned by that type of property [21]. The first two chemical steps (activation and aminoacylation) may thus contribute to a reduction of the mentioned degeneracy, a property which has been suggested earlier [5].

### Perturbation of the coding regime by mismatches

This Section discusses briefly the issue of mismatches. Let us consider the possibility that codon I is “read” by tRNA II and codon II is “read” by tRNA I. In Fig. 1(b), this implies that a red anticodon may also bind a blue codon and vice-versa. These interactions are called “mismatches”.

Let us assume that the association rate constant (*k*_+_) of these mismatches is identical to the one of complementary anticodon-codon interactions. The corresponding dissociation rate constant (*k*_−(mm)_) will usually be much higher than *k*_−(II)_ (the highest of the two dissociation rate constants of complementary interactions). Experimental data about the G·U “wobble” base-pair however suggest that these two constants could also be similar [22].

In addition to *a*, *b*, *c*, and *d* previously defined, let us define an additional parameter: *e* = *k*_−(II)_/*k*_−(mm)_. Let us now rewrite 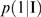 while including the additional terms due to mismatches:
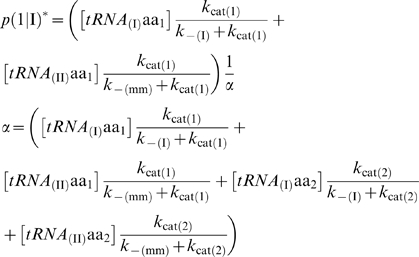
(1')


Let us consider a system in which [tRNA_(I)_] = [tRNA_(II)_] (i.e. the number of copies of the two tRNAs are identical). Following the hypothesis about the (non-selective) self-aminoacylation process described above, one has, therefore,
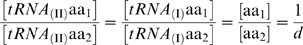
and
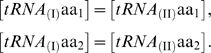
Rewriting expression (1') with the parameters *a*, *b*, *c*, *d*, *e*, one gets
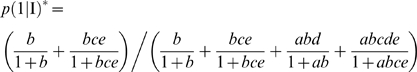
(2')Similarly, one gets for 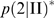:
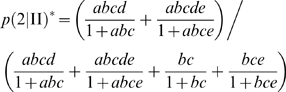
(3')


It can be verified that when *e* = 0 (i.e. the dissociation rate constant of the mismatches has a very large value), one gets the initial relations (2) and (3).

Since relations (2') and (3') cannot be further simplified, it is not straightforward to analyze Σ* with the new expressions 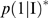 and 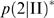. However, Σ* can be numerically examined, and it turns out that the results are rather similar to those for Σ as long as *e*≤1.

**Figure 6:**
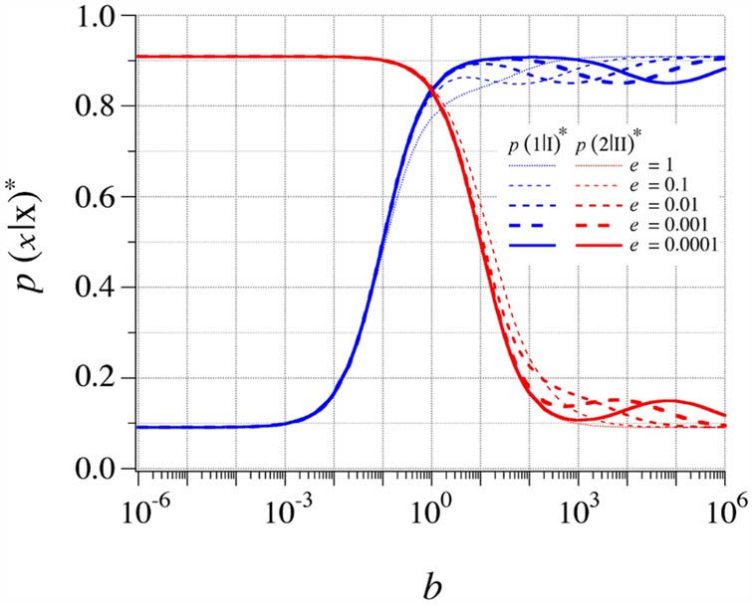
Effect of mismatches on the polymerization process. Probabilities *p*(1|I)* and *p*(2|II)* as a function of *b* for five values of *e*≤1 (*a* = 10^2^; *b* = 1; *c* = 10^−2^; *d* = 0.1). This diagram shows that the intersection between *p*(1|I)* and *p*(2|II)* (which defines the coding regime Σ*) is only slightly affected by mismatches, as long as the dissociation constant of these mismatches (*k*_−(mm)_) remains equal (*e* = 1) or higher (*e*<1) than the highest dissociation constant of the complementary matches (*k*_−(II)_).

Fig. 6 is similar to Fig. 5(a), but it includes de perturbation introduced by *e*. It can be seen that even when *k*_−(mm)_ = *k*_−(II)_ (i.e. *e* = 1), Σ* is not dramatically different from Σ, and when *k*_−(mm)_ = 10 *k*_−(II)_ (i.e. *e* = 0.1), the effect of mismatches on Σ* becomes totally negligible. Another way to examine the effect of the perturbation is to keep *b* set to 1 (with *ac* = 1), and establish the difference 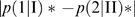 (Table 1).

**Table 1:** Perturbation of the coding regime by mismatches.

*e*	ΔΔG_0_ (kcal mol^−1^)	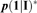	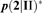	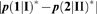
1	0	0.7739	0.8347	0.061
0.7226	0.2	0.7825	0.8434	0.061
0.1	1.417	0.8225	0.8443	0.022
0.01	2.834	0.8334	0.8360	0.003
0	very large	0.8347	0.8347	0

Numerical results with *a* = 10^2^, *b* = 1; *c* = 10^−2^, *d* = 10^−1^ (for which Σ≈0.8347). The ΔΔG_0_ indicated is the free-energy difference between the weakest complementary interaction (codon II) and the mismatch. A value of *e* = 0.7226 implies a ΔΔG_0_ of 0.2 kcal mol^−1^, which is the approximate difference between AU (Watson-Crick) and GU (Wobble) base-pairs [20].

## Discussion

Although the molecular organization of genetic code is now known in detail, there is still no agreement on the reason(s) for which it has emerged. Early studies have shown that the codon table is highly structured with respect to amino acids hydrophobicity properties, suggesting that basic physico-chemical considerations could contain the solution to this problem [21], [23]–[25]. More recent works have shown that this table is ordered with respect to features of the aminoacyl-tRNA synthetases [26] and the tRNAs [27], [28]. For instance, the mechanisms of aminoacylation as well as identity elements on the tRNAs are specific to certain groups of codons. Although these facts are fundamental, and have inspired scenarios for the evolution and the expansion of the code [26]–[28], evolutionary considerations may not, in essence, provide an answer to the origin of the code (since it is a prerequisite for biological evolution).

The present analysis shows that behind (the origin of) the code lies a problem of polymerization catalysis: how could different types of monomers (the amino acids) be involved in a same polymerization process? Whatever the exact operating mechanism(s), a single (or uniform) catalyst usually favors only one particular substrate. The four nucleotides A, G, C and U of RNA can generate conditions for the polymerization of different amino acids. In the elementary translation described here, these conditions are given by the kinetics of anticodon-codon interactions.

Among the identified requirements to set this polymerization process in the coding regime are the relative frequencies of the amino acids: the small amino acids (glycine and alanine) must be more abundant than the large ones. Although the result of Miller's experiment shown here (Fig. 2) is only indicative, it likely reflects a robust general trend which originates form the fact that complex amino acids require more chemical steps (and more energy) to be synthesized than simple ones. They are therefore expected to be less abundant, whatever the exact conditions of the environment. Our analysis thus integrates a frequency distribution which appears to be rather fundamental.

One should consider the issue of the initiation of protein synthesis in the system described here. This step is critical since a small amino acid may only weakly stabilize the initial tRNA on the ribosome cofactor. Large hydrophobic amino acids such as Leucine or Isoleucine are possible candidates since they are found in prebiotic synthesis experiments (Fig. 2). Also, the ester bond connecting these amino acids to the 3′ end of the tRNA is less prone to hydrolysis as compare with other amino acids [29], which might be critical for initiation. Another possibility is that a dipeptide already present at the 3′ end of the first tRNA [3] may help initiate translation.

In conclusion, our results show that the properties of amino acids and RNA can naturally impose a partially coded polymerization along RNA templates. We also found that the associated coding mechanism is remarkably robust against mismatches. When supplied with “meaningful” RNA sequences, translation systems of this kind should be capable of generating pools of proteins a small fraction of which will be functional. The feed-back action of these proteins on the translation itself may further increase its efficiency, allowing more codons to be added to its repertoire. In this evolutionary perspective, it can be speculated that a critical effect of emerging synthetases will be to establish only the [amino acid – tRNA] configurations that are fit for translation, a “learning” action that RNA alone cannot logically achieve.
